# Connecticut State Dental Association

**Published:** 1894-08

**Authors:** George L. Parmele

**Affiliations:** Secretary


### CONNECTICUT STATE DENTAL ASSOCIATION.

THE Thirtieth Annual Meeting of the Connecticut State Dental Association was held at Hartford, May 15 and 16. The following gentlemen were elected officers: Dr. Chas. P. Graham, of Middletown, President; Dr. Clinton W. Strang, of Bridgeport, Vice-President; Dr. George L. Parmele, of Hartford, Secretary; Dr. Daniel A. Jones, of New Haven, Treasurer.

*Executive Committee.*—Charles McManus, E. C. Barker and W. H. Rider.

Mr. Charles T. Wells, son of Horace Wells, the discoverer of anæsthesia, was unanimously elected an honorary member. The following gentlemen, having been in practice in the State over fifty years, were elected active members for life: Dr. S. J. Tuller, J. A. Pelton, and Geo. H. Waters.

Papers were read by several of the members and by Dr. Thomas C. Stellwagen, of Philadelphia, Dr. C. E. Francis, of New York, and Dr. Marfield, of Holyoke.

The Association publishes monthly a bulletin for distribution among the members.

The following resolution was introduced by Dr. Jas. McManus:

*Resolved*, That the members of the Connecticut State Dental Association, in commemorating the semi-centennial of the discovery of anæsthesia, feel it a duty to briefly restate and publish a few facts, so that the present and future generations may know the truth regarding it. In the ages past many men may have given one or more drugs with the hope of giving freedom from pain during surgical operations, but the fact is indisputable that Dr. Horace Wells, of Hartford, Connecticut, was the first man to deliberately take by inhalation an anæsthetic, and to persistently after proclaim to the world that a surgical operation had been performed on him without pain, while under the influence of nitrous oxide gas (an anæsthetic), December 11, 1844.

We would not detract from, but willingly give all due credit to, the men who have since that memorable day introduced other more powerful and dangerous anæsthetics, and which for a time were so successfully forced upon the public, by the recommendations of the medical fraternity, that the safer anæsthetic discovered by the dentist, owing to his early death, was nearly stamped out, and humanity for many years was deprived of its use and its blessings, until it was again taken up by Connecticut dentists, and its value as a safe anæsthetic proved daily since 1863 in thousands of operations.

The physicians and surgeons of the city of Hartford united in a testimonial, declaring their belief in the justice of the claims of Dr. Wells, in 1845–46.

The General Assembly of Connecticut, in 1847, passed resolutions in favor of Dr. Wells, the discoverer of anæsthesia, and declared that he was entitled to the favorable consideration of his fellow-citizens, and to the high credit of a public benefactor.

The Court of Common Council of the city of Hartford passed resolutions to the same effect.

In France, the medical society in Paris, January, 1848, voted: "To Dr. Horace Wells, of Hartford, Connecticut, is due all the honor of having first discovered anæsthesia."

The State of Connecticut and the city of Hartford, with the added contributions from citizens of this and other States, have had placed on Bushnell Park, in Hartford, a portrait statue in bronze of Dr. Horace Wells; and the past year the Board of Dental Commissioners have had stamped on their certificates of registration and license a seal, on which is a medallion head of Dr. Wells, to remind all dentists in this state of the honor due to his memory.

The public should not be allowed to forget that the simple, honest, Christian desire of this dentist was to give his discovery to all to be "free as the air we breathe," and his course in regard to it stands out grandly when compared with the extortionate plans laid by all other vendors of secret anæsthetics, for the use of which they demand of suffering humanity an unjust tribute.

Drs. Geo. L. Parmele, Jas. McManus and Civilion Fones have been appointed a committee to arrange for the semi-centennial celebration. It is proposed to have a banquet, and to place a memorial tablet to mark the spot where the first operation under an anæsthetic was performed.

GEORGE L. PARMELE, D.M.D.,
*Secretary.*

